# Pressurized intraperitoneal aerosol chemotherapy (PIPAC) for rare gynecologic indications: peritoneal metastases from breast and endometrial cancer

**DOI:** 10.1186/s12885-020-07627-1

**Published:** 2020-11-19

**Authors:** Günther A. Rezniczek, Urs Giger-Pabst, Omar Thaher, Clemens B. Tempfer

**Affiliations:** 1 Department of Obstetrics and Gynecology, Marien Hospital Herne, Ruhr-Universität Bochum, Hölkeskampring 40, Herne, 44625 Germany; 2 Department of Surgery, University of Münster, Münster, Germany; 3 Department of Surgery, Marien Hospital Herne, Ruhr-Universität Bochum, Herne, Germany

**Keywords:** PIPAC, Peritoneal metastasis, Breast cancer, Endometrial cancer, Tumor regression

## Abstract

**Background:**

Peritoneal metastasis (PM) in patients with breast (BC) and endometrial cancer (EC) is rare and treatment options are limited. Pressurized IntraPeritoneal Aerosol Chemotherapy (PIPAC) has demonstrated efficacy against PM from various cancers, but its efficacy in BC/EC patients is unknown.

**Methods:**

Retrospective cohort study of patients with PM from BC/EC undergoing PIPAC with doxorubicin 1.5 mg/m^2^ and cisplatin 7.5 mg/m^2^. Data were collected within an international prospective PIPAC registry. Study outcomes were microscopic tumor regression grade (TRG), survival, adverse events (CTCAE), and quality of life (QoL).

**Results:**

150 PIPAC procedures in 44 patients (BC/EC = 28/16; mean age 58.8 ± 10.1 and 63.2 ± 10.1 years, respectively) were analyzed. The mean number of PIPACs per patient was 3 (range 0–9) and 3.5 (range 0–10), respectively. Primary/secondary non-access occurred in 4/3 of 150 (5%) procedures. PIPAC induced objective tumor regression as demonstrated by repetitive PM biopsies in 73% (32/44) of patients. Peri- and postoperative CTCAE grade 3 and 4 complications were observed in 12/150 (8%) of procedures. No grade 5 event was observed. After a median follow up of 5.7 (IQR 2.7–13.0) months, overall median survival was 19.6 (95% CI: 7.8–31.5) months (from first PIPAC). QoL indicators (general health, nausea, fatigue, constipation, pain, dyspnea, social, cognitive, emotional, and physical functioning) all improved or were maintained throughout PIPAC treatments.

**Conclusions:**

Repetitive intraperitoneal chemotherapy with PIPAC is feasible and safe in patients with PM from BC and EC. PIPAC induces significant histological regression of PM while maintaining QoL.

## Background

Peritoneal metastasis (PM) in patients with breast cancer (BC) and endometrial cancer (EC) is a rare and challenging condition. Patients with PM account for less than 3% of recurrent BC and EC cases [1–4]. In a comprehensive review of the literature, only 21 articles with 505 patients with BC and PM were identified [1]. Based on these data, PM was associated with invasive lobular histology, loss of functional p53, and loss of E-cadherin expression. BC patients with PM have a very poor prognosis. For example, in a retrospective series of 44 patients, the median survival from the diagnosis of BC and PM was only 1.5 months (range 0.2–27 months) [2]. In analogy to patients with BC and PM, patients with EC and PM also have a poor prognosis [5, 6]. In a French multicenter study with 1230 EC patients, for example, metachronous PM developed in less than 2% of cases [7]. Patients with PM had significantly shorter survival times compared to EC patients with other recurrence pathways. In accordance, Ozkan et al. found that the 5-year progression-free survival rate of patients with EC and PM was 35% compared to 54% for patients with EC and vaginal vault recurrence [8].

Pressurized IntraPeritoneal Aerosol Chemotherapy (PIPAC) is a new technique to deliver intraperitoneal chemotherapy with the aim of achieving high local concentrations of chemotherapy compounds in the peritoneum [9–13]. Recently, Alyami et al. summarized 45 clinical studies with 1810 PIPAC procedures in 838 patients [14]. In this review, repeated PIPAC was feasible in 64% of patients with a 3% rate of intraoperative and postoperative surgical complications. Objective histological response was 62 to 88% for ovarian cancer, 50 to 91% for gastric cancer, and 71 to 86% for colorectal cancer. In this comprehensive review, however, no data on PIPAC in patients with BC and EC were identified. In addition, we performed a PubMed search (search terms: PIPAC, intraperitoneal chemotherapy, pressurized intraperitoneal chemotherapy, breast cancer, endometrial cancer; search date: April 8, 2020) and confirmed that there are no studies available reporting on PIPAC in patients with BC or EC.

During the last 7 years we established a comprehensive PIPAC program at our institution with clinical experience in > 1000 PIPAC procedures including patients with BC and EC [15]. In order to address the feasibility, safety, and efficacy of PIPAC in patients with BC and EC, we identified all cases of PM from BC and EC treated with PIPAC in our institution and herein report the clinical results.

## Methods

### Patients and regulatory framework

The regulatory framework and patient selection criteria for PIPAC have been described previously [16]. Patient and procedure data were collected within a prospective PIPAC registry approved by the local Institutional Review Board (Ethics Committee of the Ruhr-Universität Bochum, Germany; registration number 15–5280). Furthermore, the present retrospective cohort study including PIPAC patients with BC and EC was approved by the same review board (registration number 19–6612).

### PIPAC procedure

The standard PIPAC procedure has been previously described in detail [15]. In brief, access to the abdominal cavity was obtained via a Verres needle inserted at Palmer’s point in the left upper abdomen. If possibe, peritoneal biopsies were retrieved from all four abdominal quadrants and sent for histological analysis. The standard drug regimen for gynecologic cancers used for aerosolization: doxorubicin (1.5 mg/m^2^ body surface in 50 ml NaCl 0.9%) followed by cisplatin (7.5 mg/m^2^ body surface in 150 ml NaCl 0.9%). After the publication of a phase I dose-escalation trial of PIPAC we have changed the dosage to doxorubicin 2.1 mg/m^2^ body surface and cisplatin 10.5 mg/m^2^ body surface [17]. Senior surgeons trained in PIPAC performed all procedures.

### Data collection, follow-up, and statistical analysis

Data collection and follow-up procedures were as previously described [16]. In brief, patient and procedure data were collected into a prospective PIPAC registry, including follow-up data. The last follow-up date was April 8, 2020. EORTC QLQ-C30 (Version 3.0) was used to assess quality of life (on the day before each PIPAC). Histological tumor response was assessed by the Institute of Pathology, Ruhr-Universität Bochum, Bochum, Germany. To evaluate the histological tumor regression grade (TRG) induced by PIPAC, the following criteria according to Dworak et al. were applied: TRG 0 = no regression; TRG 1 = some signs of regression (tumor with obvious fibrosis with/without vasculopathy); TRG 2 = strong signs of regression (significant fibrotic changes with few scattered tumor cells or groups in the space of fibrosis with/without acellular mucin); and TGR 3 = no vital tumor cells detectable [18]. Adverse events were graded according to the Common Terminology Criteria for Adverse Events (CTCAE, v4.03) [19]. The relatedness (not related, possibly related, related to PIPAC) was not systematically assessed and AEs were not compared to baseline symptoms. AE assessment was done until 3 days after each PIPAC and during each PIPAC cycle.

Study data were collected and managed using REDCap (Research Electronic Data Capture), a secure web-based application designed to support data capture for research studies [20]. After data collection, exported data were further processed in Microsoft Excel (Microsoft Inc., Redmond, WA) and prepared for statistical analyses using SigmaPlot 14 (Systat Software Inc., San Jose, CA). Categorical data are given as absolute numbers (percentage proportions), continuous data as means (standard deviations) or medians (interquartile ranges), as appropriate, depending on the results of the Shapiro-Wilk normality test. Overall median survival was modelled using a Kaplan-Meier curve, using the date of the first PIPAC treatment as the start date. Figure plots were generated using SigmaPlot and final figures were assembled in Illustrator (Adobe Inc., Palo Alto, CA).

## Results

### Patient characteristics

Between August 2013 and November 2019, a total of 44 patients (BC/EC = 28/16) with a mean age of 58.8 ± 10.1 and 63.2 ± 10.1 years, respectively, were analyzed. All patients had histologically proven PM from BC/EC. Histologically or radiologically confirmed extra-abdominal metastases prior to the first PIPAC were observed in 21/28 (BC) and in 3/16 (EC) patients, respectively. Concurrent systemic oncological therapy was performed in 23/28 patients with BC (mostly bisphosphonates and systemic chemotherapy) and in 7/16 patients with EC (mostly endocrine therapy with progestins). Patients were heavily pretreated. The mean number of prior major surgeries was 3 (range 1–9) in patients with BC and 1.5 (range 1–3) in patients with EC, respectively. Specifically, patients underwent 37 breast surgeries (31%), 7 hysterectomies (with or without lymphadenectomy) (14%), 22 major abdominal surgeries (19%), and 43 other surgeries (36%). The mean number of prior systemic chemotherapy lines was 2 (range 0–8) and 1 (range 0–2), respectively. Further details of patient and tumor characteristics are given in Table 1.

**Table 1:** Baseline characteristics of patients and their primary cancers

Characteristic / Data Item	Primary Tumor Entity	
	Breast Cancer	Endometrial Cancer
Total number of patients (*N* = 44)	28	16
Age, y	58.8 ± 10.1 (range 33–80)	63.2 ± 10.1 (range 47–80)
Body mass index, kg/m^2^	25.3 ± 5.7	27.7 ± 2.8
Smoking (yes / no)	9 / 18 [1]	5 / 9 [2]
Allergies (yes / no)	9 / 19	5 / 11
Concomitant diseases	2 (0.25–4), range 0–6	2 (1–4), range 0–5
Prior major surgeries	3 (2–4), range 1–9	1.5 (1–2), range 1–3
ECOG performance score		
*ECOG 0*	9 (32.1%)	7 (43.8%)
*ECOG 1*	18 (64.3%)	9 (56.8%)
*ECOG 2*	1 (3.6%)	–
Karnofsky index		
*100%*	1 (3.6%)	3 (18.8%)
*90%*	8 (28.6%)	4 (25.0%)
*80%*	14 (50.0%)	6 (37.5%)
*≤ 70%*	5 (17.8%)	3 (18.8%)
Histological type		[1]
*Lobular invasive*	7 (25.0%)	–
*Ductal invasive*	18 (64.3%)	–
*Inflammatory*	1 (3.6%)	–
*Other*	2 (7.1%)	–
*Endometrioid adenocarcinoma*	–	5 (33.3%)
*High-grade adenocarcinoma*	–	8 (53.3%)
*Serous*	–	1 (6.7%)
*Clear cell*	–	1 (6.7%)
Tumor stage		
*T1*	12 (42.9%)	11 (68.8%)
*T2*	9 (32.1%)	1 (6.3%)
*T3 / T4*	7 (25.0%)	4 (25.0%)
*N0*	9 (32.1%)	4 (25.0%)
*N1 / N2*	18 (64.3%)	2 (12.5%)
*NX*	1 (3.6%)	10 (62.5%)
Metastases outside the peritoneum at the time of first PIPAC (yes / no)	21 / 7	3 / 13
*Bone*	14 (50.0%)	–
*Liver*	3 (10.7%)	2 (12.5%)
*Pleura*	5 (17.9%)	–
*Other*	7 (25.0%)	1 (6.3%)
Systemic oncological therapy concurrent with PIPAC (yes / no)	23 / 5	7 / 9
*Chemotherapy*	4 (14.3%)	–
*Endocrine therapy*	12 (42.9%)	7 (43.8%)
*Bisphosphonates/denosumab*	11 (39.3%)	–
*Other*	2 (7.1%)	–
Prior chemotherapy lines	2, range 0–8	1, range 0–2
Type of chemotherapy		
*Anthracycline*	11 (39.3%)	–
*Platinum*	2 (7.1%)	–
*Taxane*	8 (28.6%)	1 (6.3%)
*Anthracycline + platinum*	–	3 (18.8%)
*Anthracycline + taxane*	9 (32.1%)	2 (12.5%)
*Platinum + taxane*	3 (10.7%)	8 (50.0%)
*Other*	10 (35.7%)	1 (6.3%)
Targeted therapy (yes / no)	8 / 20	2 / 14
*Bevacizumab*	3 (10.7%)	2 (12.5%)
*Trastuzumab*	4 (14.3%)	–
*Pertuzumab*	1 (3.6%)	–
*Trastuzumab + pertuzumab*	1 (3.6%)	–
*Lapatinib*	1 (3.6%)	–
Immune checkpoint inhibitor (yes / no)	1 / 28	0 / 16
*Palbociclib*	1 (3.6%)	–

Values are counts (percentage proportions; subgroup percentages are relative to the group; due to rounding, sums may not add up to 100%), means ± standard deviations, medians (interquartile ranges), or medians and range. Numbers in square brackets indicate the number of missing values. *ECOG* Eastern Cooperative Oncology Group performance status, *PIPAC* Pressurized intraperitoneal aerosol chemotherapy

### PIPAC applications

PIPAC was feasible in the study population. In summary, a total of 150 PIPAC procedures (BC/EC = 82/68) were performed. The mean number of PIPACs per patient was 3 (range 0–9) in patients with BC and 3.5 (range 0–10) in patients with EC. Primary/secondary non-access occurred in 4/3 of 150 (5%) procedures. One PIPAC, 2–3 PIPACs, 4–6 PIPACs, and 7–10 PIPACS were performed in 12, 10, 13, and 5 patients, respectively. More procedural details of all 150 PIPACs are given in Table 2.

**Table 2:** PIPAC procedures

Characteristic / Data Item	Primary Tumor Entity	
	Breast Cancer	Endometrial Cancer
Number of patients (*N* = 44)	28	16
Total number of procedures (*N* = 150)	82	68
*Completed procedures*	78 (95.1%)	62 (91.2%)
Completed PIPACs per patient	3 (1–4), range 0–9	3.5 (1–6.75), range 0–10
*7–10 procedures*	1 (3.6%)	4 (25.0%)
*4–6 procedures*	9 (32.1%)	4 (25.0%)
*2 or 3 procedures*	7 (25.0%)	3 (18.8%)
*1 procedure*	10 (35.7%)	2 (12.5%)
*0 procedures*	1 (3.6%)	3 (18.8%)
Primary non-access	1 (1.3%)	3 (4.8%)
Secondary non-access	1 (1.3%)	2 (3.2%)
RECIST		
*Complete remission*	3 (10.7%)	–
*Partial remission*	9 (32.1%)	4 (25.0%)
*Stable disease*	3 (10.7%)	2 (12.5%)
*Disease progression*	–	1 (6.3%)
*Not determined / too few PIPACs*	13 (46.4%)	9 (56.3%)
Overall peri−/postoperative morbidity	1 (0–2), range 0–4	1 (0–2), range 0–4
*Total number of adverse events*	83	68
*Procedures without complications*	34 (40.9%)	28 (41.2%)
*CTCAE grade 1*	21 (25.3%)	21 (30.9%)
*CTCAE grade 2*	19 (22.9%)	16 (23.5%)
*CTCAE grade 3*	7 (8.4%)	3 (4.4%)
*CTCAE grade 4*	2 (2.4%)	–
Overall peri−/postoperative mortality	0	0

Values are counts (percentage proportions; due to rounding, sums may not add up to 100%) or medians (interquartile ranges). Peri−/postoperative morbidity data give the median (interquartile range) and range of complications per procedure and the number (proportion) of procedures where the highest indicated CTCAE grade occurred. *CTCAE* Common Terminology Criteria for Adverse Events (Version 4.03), *PIPAC* Pressurized intraperitoneal aerosol chemotherapy, *RECIST* Response evaluation criteria in solid tumors

### Adverse events and toxicity

During 150 PIPAC procedures, 33/44 patients (75%) experienced a total of 151 complications (CTCAE grades 1–4). The median number of complications per patient was 1 (range 0–4). Mild postoperative complications (CTCAE grade 1) such as abdominal pain, anemia, nausea/vomiting, dyspnea, urinary tract infection, and appetite loss were frequent and occurred in 50, 1, 31, 7, 1, and 1 cases, respectively. CTCAE grade 2 events comprised abdominal pain (*N* = 20), anemia (*N* = 3), dyspnea (*N* = 2), nausea (*N* = 3), pleural effusion (*N* = 2), trocar wound complication (*N* = 1), and thrombosis (*N* = 2). Twelve severe complications CTCAE grade 3/4 occurred in 150 procedures (8.0%), affecting 8 of 44 patients (details are given in Table 3) The overall mortality rate of all included patients was 0% (0/44). The procedure-related mortality rate was 0% (0/150). Details of all postoperative adverse events are summarized in Table 3.

**Table 3:** Peri−/postoperative morbidities according to CTCAE

Adverse Events	CTCAE Grade				
	1	2	3	4	5
Abdominal pain	50	20	–	–	–
Anemia	1	3	–	–	–
Appetite loss	1	–	–	–	–
Bowel obstruction	–	–	1	1	–
Cachexia	1	–	–	–	–
Cutaneous abscess	–	–	1	–	–
Diarrhea	–	–	2	–	–
Dyspnea	7	2	1	–	–
Fatigue	1	–	–	–	–
Nausea	31	3	–	–	–
Pleural effusion	–	2	1	–	–
Pneumonia	–	–	2	–	–
Pulmonary embolism	–	–	1	–	–
Exacerbation of radiation proctitis	–	–	1	–	–
Small bowel perforation ^a^	–	–	–	1	–
Trocar wound complications	4	1	–	–	–
Tachycardia	–	1	–	–	–
Thrombosis (lower extremity)	–	2	–	–	–
Urinary tract infection	1	2	–	–	–
Other	5	2	–	–	–
Total (*N* = 151)	103	36	10	2	0

Values are counts. *CTCAE* Common Terminology Criteria for Adverse Events (Version 4.03). ^a^One case of small bowel perforation during laparoscopic PIPAC access occurred. The lesion was recognized and repaired via mini-laparotomy and PIPAC was resumed

**Fig. 1:**
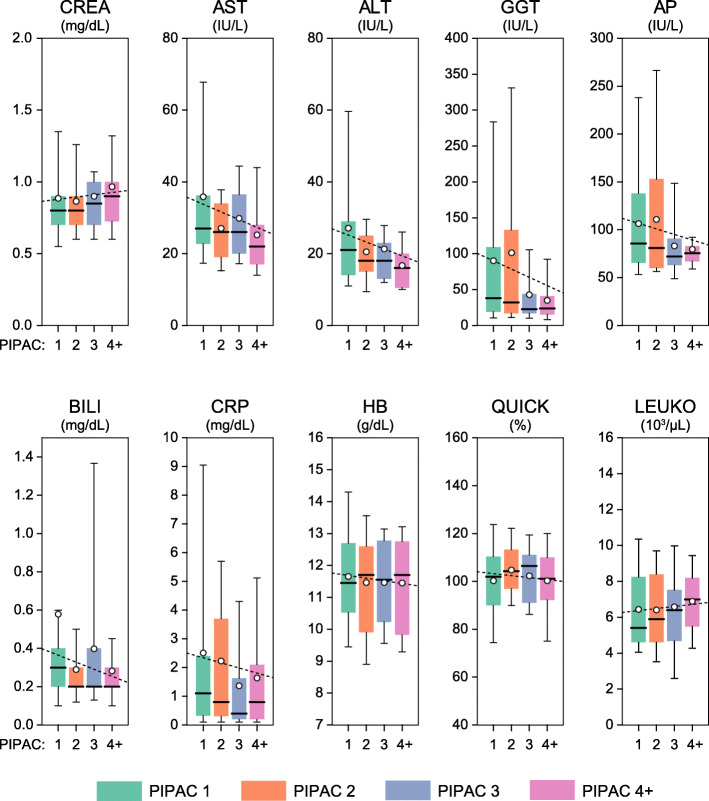
Blood parameters at baseline (PIPAC 1) and during PIPAC treatment cycles 2, 3, and 4+, respectively (*N* = 40–44, 30–31, 20–22, and 43–48; respectively). CREA, creatinine; AST, aspartate transaminase; ALT, alanine aminotransferase; GGT, γ-glutamyl transferase; AP, alkaline phosphatase; BILI, bilirubin; CRP, c-reactive protein; HB, hemoglobin; LEUKO, leukocytes; QUICK, Quick‘s test. Box plots: lower/upper boundaries of boxes represent the 25th/75th percentiles, thick lines the medians, and the whiskers the 10th and 90th percentiles, respectively. Open circles represent the means, and dotted lines the linear regression through all individual data points

Repetitive PIPACs did not induce systemic toxicity. Specifically, c-reactive protein, blood parameters such as hemoglobin and hematocrit, coagulation parameters as well as renal and hepatic functional parameters were monitored in all patients throughout PIPAC cycles. Figure 1 demonstrates that all markers of systemic toxicity were stable during the course of PIPACs 1, 2, 3, and ≥ 4.

### Tumor regression assessment, ascites & survival

**Fig. 2:**
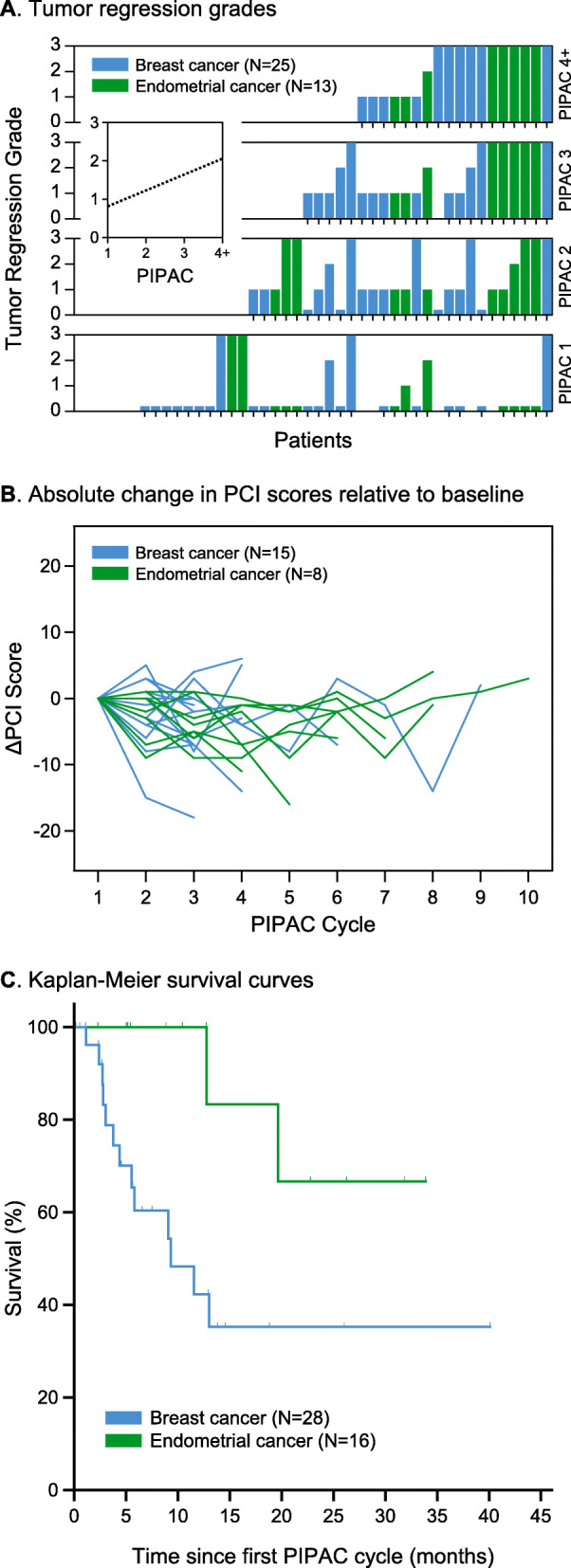
**a** Tumor regression induced by PIPAC treatments. Bars indicate tumor regression in individual patients undergoing PIPAC cycles 1, 2, 3, or 4 and more (each column across the panels represents an individual patient). Inset with dotted line: linear regression (*p* < 0.001, regression coefficient 0.416). Tumor regression grade 0 to 3, based on Dworak et al.: 0, no regression; 1, some regression; 2, major regression; 3, complete regression. **b** Spaghetti plot of peritoneal carcinomatosis index (PCI) scores. The PCI score at baseline (PIPAC 1) was used as reference and absolute increase/decrease of PCI score during PIPAC cycles is shown. Each line represents a patient for whom at least three data points were available. **c** Kaplan-Meier survival analysis. In all panels, data from patients with primary breast cancer are shown in blue and those from patients with primary endometrial cancer are shown in green

At least one peritoneal biopsy from a representative area suggestive of PM was available from 132/150 PIPACs and were sent for histological regression analysis. No tumor regression (TRG 0) was observed in 30/132 procedures (22.7%). TRG 1, TRG 2, and TRG 3 were observed in 49/132 (37.1%), 10/132 (7.6%), and 43/132 (32.6%), respectively. On an intention-to-treat basis, PIPAC thus caused histological tumor regression (TRG > 0) in 73% of patients (32/44). Comparison of TRG scores between consecutive PIPAC cycles showed a significant increase in TRG during PIPAC applications (*p* < 0.001; regression coefficient = 0.416). TRG 2/3 was observed in 21/38 patients (55.3%) who underwent at least one PIPAC treatment and where TRG was assessed. Figure 2a demonstrates the histological TRG induced by PIPAC broken down by patient and number of PIPACs.

The proportion of patients with malignant ascites significantly decreased during consecutive PIPAC applications (*p* = 0.049). Less obvious results were observed for the PCI score, which improved or was maintained during consecutive PIPAC applications as demonstrated in Fig. 2b.

The follow up period started with the first PIPAC application. During a median follow up period of 5.7 (IQR 2.7–13.0; range 0.1–40.1; mean 9.8) months, overall median survival was 19.6 (95% CI: 7.8–31.5) months [mean: 22.5 (95% CI: 15.9–29.0) months], from the date of the first PIPAC treatment. At the end of the observation period, a total of 15 patients (13/28 BC, 2/16 EC) had died. Survival data of BC and EC patients separately are given in Fig. 2c. Mean overall survival was 18.3 (95% CI: 10.5–26.0) months for BC (median: 9.3 months) and 28.0 (95% CI: 18.3–37.6) months for EC patients.

### Quality of life

**Fig. 3:**
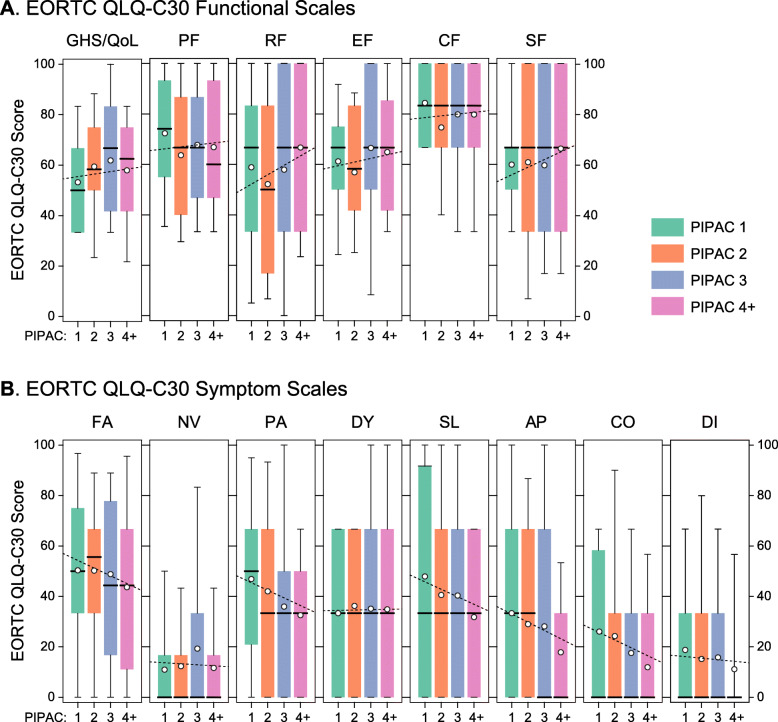
Quality of Life scores according to the EORTC QLQ-C30 questionnaire at baseline (PIPAC 1; *N* = 32) and during PIPAC treatment cycles 2 (*N* = 23), 3 (*N* = 19), and 4+ (*N* = 43), respectively. **a** Functional scores: GHS/QoL, global health score, quality of life; PF, physical functioning; RF, role functioning; EF, emotional functioning; CF, cognitive functioning; SF, social functioning. **b** Symptom scales: FA, fatigue; NV, nausea and vomiting; PA, pain; DY, dyspnea; SL, insomnia; AP, appetite loss; CO, constipation; DI, diarrhea. Box plots: lower/upper boundaries of boxes represent the 25th/75th percentiles, thick lines the medians, and the whiskers the 10th and 90th percentiles, respectively. Open circles represent the means, and dotted lines the linear regression through all individual data points

Quality of life was maintained or improved during repetitive PIPACs (data available from 32/44 patients). Specifically, the functional scales global health score, physical functioning, role functioning, emotional functioning, social functioning, and cognitive functioning continuously improved during repetitive PIPACs (Fig. 3a). In accordance, the symptom scales for fatigue, pain, appetite loss, constipation, and diarrhea decreased over time, whereas the symptom scales for nausea/vomiting and dyspnea remained constant (Fig. 3b).

## Discussion

PM in patients with BC and EC is associated with a poor prognosis and treatment options for this condition are limited. In a retrospective cohort study of patients with PM from BC and EC undergoing 150 PIPACs with doxorubicin and cisplatin, we found that this treatment was feasible and induced objective TRG in up to 55% of patients while maintaining quality of life. PIPAC induced no systemic toxicity and treatment-associated morbidity was acceptable with CTCAE grade 3/4 in 8% of procedures.

PIPAC is a new form of intraperitoneal chemotherapy for patients with PM from various origins, including BC and EC, where cytostatic drugs are laparoscopically delivered in the form of a pressurized aerosol rather than in liquid form during conventional intraperitoneal chemotherapy. This is thought to offer a number of advantages, supported by experimental evidence: increased surface/volume ratio of the aerosol droplets versus larger liquid volumes, better diffusion and influx by convection, as well as enhanced tissue penetration under pressure [21, 22]. Furthermore, PIPAC can be applied repeatedly, providing the opportunity to obtain sequential tumor biopsies and PCI assessment, thus allowing the objective monitoring of therapy response. Safety and feasibility of PIPAC have been demonstrated in a number of studies on patients with mesothelioma, ovarian, gastric, pancreatic and colorectal cancer [10, 12–15, 23–25]. We now confirm, for the first time, that PIPAC is also feasible and safe in patients with PM from BC and EC.

In our series, we encounterd a primary and/or secondary non-access of 5%, which is comparable to that observed by other groups [10, 12, 26, 27]. The rate of postoperative complications (12 CTCAE grade 3/4 events in 150 PIPACs, some of which were unlikely to be procedure-related) was acceptable. Since the deepest tissue penetration, as demonstrated by ex-vivo studies [28, 29], occurs in the small bowel, we did not perform adhesiolysis during PIPAC in order to avoid small bowel laceration. This might be one reason why there were no abdominal adverse events secondary to PIPAC.

Objective tumor regression was seen in > 70% of our patients with PM and major regression was achieved in more than 50% of patients. Matching up these findings with systemic chemotherapy outcomes reported by previous studies [1–4] suggests PIPAC as a suitable therapy alternative. In addition, 30/44 patients had concomitant systemic chemotherapy or endocrine therapy, suggesting that PIPAC is also suitable in this patient population for a bidirectional therapy approach. This finding is in line with a recent report by Ploug et al. confirming the feasibility of bidirectional PIPAC and systemic chemotherapy in non-gynecological cancer patients [27].

We found that PIPAC may preserve quality of life, as most functional scales such as the global health score, physical functioning, role functioning, emotional functioning, social functioning, and cognitive functioning had increased over time, while symptom scales of gastrointestinal toxicity (appetite loss, constipation, and diarrhea) were decreased. This is of note in such a population of patients with BC and EC, diagnosed with PM, that is treated with palliative intent and underscores the potential validity of PIPAC in such a setting.

Our study has limitations. The sample size is small, but this is usually the case when reporting on a rare disease such as PM in patients with BC and EC. As this is the first report on PIPAC in patients with BC and EC, our results must be independently confirmed. Furhtermore, the retrospective design of this study limits the internal validity of our study. Selection bias may have played a role because only fit patients were eligible for this therapy. Self-selection of patients may also have affected the results in favor of PIPAC. In addition, patients abandoning the therapy because of side effects or weakness also contribute to selection. However, these limitations also apply to studies evaluating palliative systemic chemotherapy. Prospecitve studies that avoid these limitations by applying uniform inclusion criteria should be carried out.

## Conclusions

Treatment with repetitive cycles of PIPAC in patients with BC and EC metastasizing to the peritoneum is feasible and safe and may preserve quality of life. PIPAC induced objective tumor regression in up to 55% of patients.

## Data Availability

The dataset and/or analyzed during the current study are available from the corresponding author on reasonable request.

## Abbreviations

BC: Breast cancer
CI: Confidence interval
CRS: Cytoreductive surgery
CTCAE: Common Terminology Criteria for Adverse Events
EC: Endometrial cancer
EORTC: *European Organisation for Research and Treatment of Cancer*
ECOG PS: Eastern Cooperative Oncology Group performance score
HIPEC: Heated intraperitoneal chemotherapy
IPC: Intraperitoneal chemotherapy
IQR: Interquartile range
KI: Karnofsky index
PCI: Sugarbaker’s peritoneal carcinomatosis index
PIPAC: Pressurized intraperitoneal aerosol chemotherapy
PM: Peritoneal metastasis
QoL: Quality of life
TRG: Tumor regression grade
